# Efficacy of a high-intensity home stretching device and traditional physical therapy in non-operative management of adhesive capsulitis - a prospective, randomized control trial

**DOI:** 10.1186/s12891-024-07448-4

**Published:** 2024-04-20

**Authors:** David E. Teytelbaum, Neil S. Kumar, Craig S. Dent, Spencer Neaville, Deborah H. Warren, Peter Simon, Christopher E. Baker

**Affiliations:** 1 Foundation for Orthopaedic Research and Education, Tampa, FL USA; 2 Florida Orthopaedic Institute, 13020 Telecom Parkway North, Temple Terrace, Tampa, FL 33637 USA; 3 Department of Medical Engineering, College of Engineering and Morsani College of Medicine, University of South Florida, Tampa, FL USA

**Keywords:** Adhesive capsulitis, Physical therapy, High-intensity stretching device

## Abstract

**Background:**

Historically, in-person physical therapy serves as a foundational component of nonoperative treatment of adhesive capsulitis (AC). This study compares the effectiveness of an at-home high-intensity stretch (HIS) device to traditional physical therapy (PT) and to PT in combination with the HIS device. We hypothesize that the HIS device will be as effective as PT alone or as combination therapy in the first-line treatment of AC and use of the HIS device will exhibit improvement at higher rate.

**Methods:**

Thirty-four patients with idiopathic adhesive capsulitis and a minimum of 12 months follow-up were included in this study. Patients were randomized into one of the three groups: HIS device, PT alone, or HIS device + PT. Passive range of motion (ROM), American Shoulder and Elbow Surgeons (ASES), and Simple Shoulder Test (SST) scores were measured. Additionally, patient satisfaction, compliance and complications were recorded. Paired t-test, ANOVA and Chi-squared tests were used in analysis.

**Results:**

Final ROM in all planes improved for all groups compared to baseline (*p* < 0.001), with only HIS device group able to restore > 95% of contralateral ROM in all planes at final follow-up. Patients with PT alone were on average slowest to improve ROM from baseline, at 3 months, 6 months, and 1 year in all planes except internal rotation. ASES and SST scores improved for all groups when compared to baseline (*p* < 0.001). Use of HIS-device resulted in greater improvement in SST and ASES Total scores compared to PT alone (*p* = 0.045, and *p* = 0.048, respectively).

**Conclusions:**

Use of an at-home high-intensity stretching device for conservative treatment of idiopathic adhesive capsulitis improves outcomes in ROM and in ASES and SST scores both when used as an adjunct to physical therapy and when used alone.

**Trial registration:**

The study protocol was registered at www.clinicaltrials.gov (20/05/2022, NCT05384093).

**Supplementary Information:**

The online version contains supplementary material available at 10.1186/s12891-024-07448-4.

## Background

Idiopathic adhesive capsulitis (AC), commonly referred to as “frozen shoulder,” is a debilitating condition resulting in painful and restricted range of motion (ROM) due to shoulder joint stiffness. Its incidence in the United States has been reported between 2–5% [1, 2]. Although the etiology is largely unknown, it has been associated with diabetes mellitus, thyroid dysfunction, and autoimmune disease [3–7]. All planes of motion are commonly affected in AC, although passive external rotation is typically more limited than abduction or internal rotation [8].

Treatment of AC is aimed at relieving pain and restoring motion and function of the shoulder. Conservative therapy commonly includes oral nonsteroidal anti-inflammatory drugs (NSAIDs), intra-articular steroid injections, and physical therapy (PT) [2, 3, 9]. Surgical treatment options include manipulation under anesthesia with or without capsular release, commonly performed arthroscopically [2, 3, 9]. Physical therapy is the most prescribed treatment for AC. Despite its widespread use, there is a lack of high-level evidence supporting the use of PT for the treatment of AC [10–12]. Griggs et al. evaluated 75 patients with AC treated with a stretching program and found 90% achieved a satisfactory outcome [13]. A meta-analysis by Jewell et al. found joint mobilization and exercises were the most effective modality for AC [14, 15].

There are significant costs regarding disability due to adhesive capsulitis and its management. Direct costs of managing AC were approximately $7 billion in the United States in 2000, and reached to greater than $9 billion in 2017 [16]. Absence from work, difficulty with sleep, and inability to perform activities of daily living can be a significant burden to patients. Visits to physician offices and PT appointments can lead to significant financial and time costs for patients. Providing an efficient and cost-effective treatment plan continues to be a challenge in the treatment of AC [17]. Mechanical therapy performed at home has been a successful adjunct to outpatient PT for adhesive capsulitis because of its unique ability to apply torque, similar to a physical therapist, to stiff joints [16, 18–21]. Patients typically are given high-intensity stretch (HIS) devices when they are not meeting treatment milestones and have reached a plateau in their recovery using standard physical therapy. Further, improvements in ROM can be achieved in patients regardless of pre-interventional irritability level [22, 23]. However, HIS devices have not been studied as first-line therapy in the treatment of AC.

This study aimed to evaluate and compare the efficacy of AC treatment therapies. We further aimed to compare the rate of improvement within studied therapy options. And lastly, we reported on patient’s compliance and overall satisfaction with HIS device. We hypothesize that the HIS device will be as effective as PT alone or as combination therapy in the first-line treatment of AC and HIS device will exhibit improvement at higher rate.

## Methods

### Study settings

This study is a prospective randomized control study beginning in September of 2019 until December 2022. All patients in the study were treated by one of two fellowship-trained orthopaedic surgeons at the Florida Orthopaedic Institute in Tampa, FL (CB, NK) prior to randomization into therapy.

### Participants

**Fig. 1:**
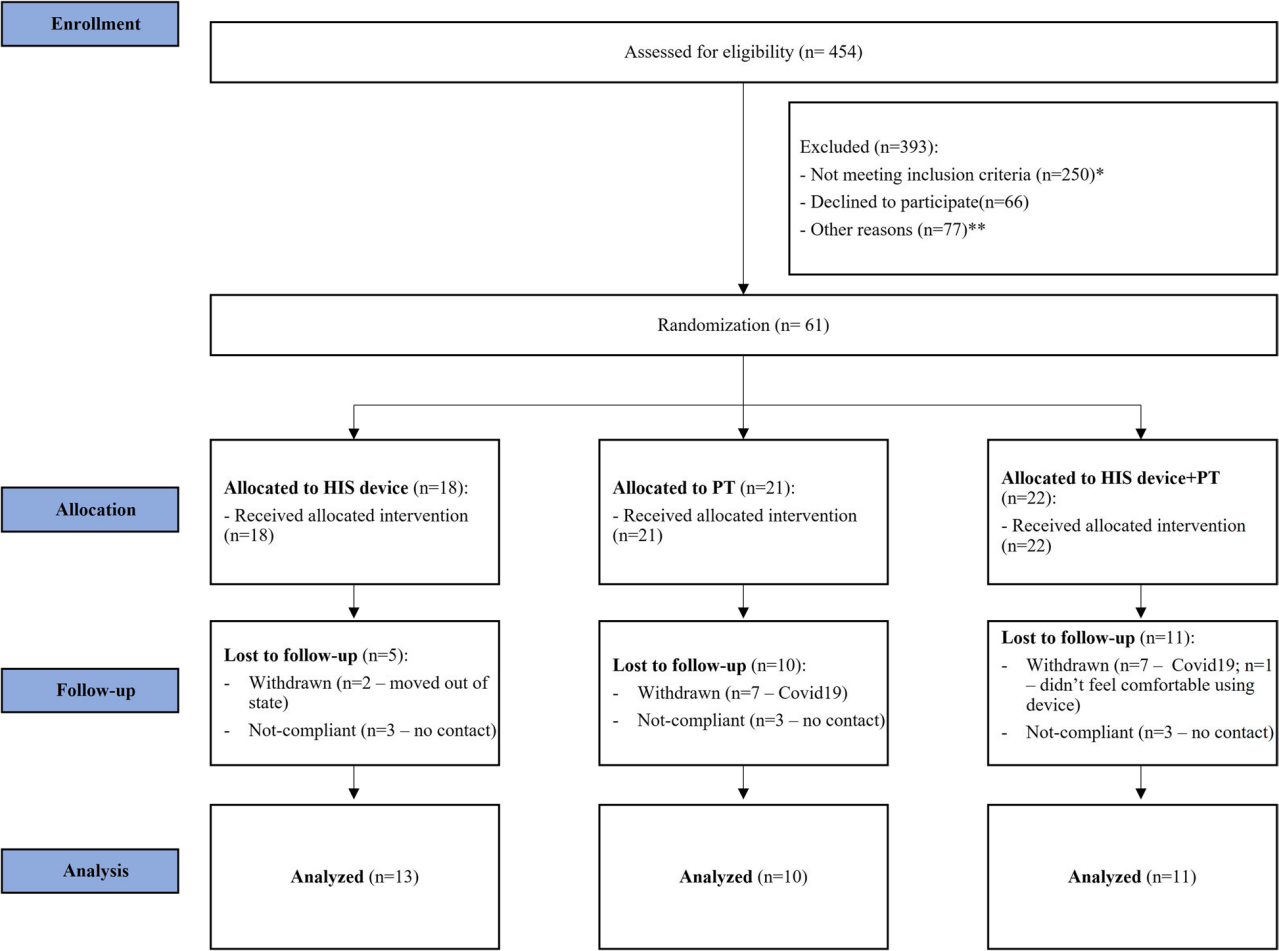
Patient screening and eligibility with subsequent enrollment. * Not meeting inclusion criteria: a) 26% (118 patients) had superior ROM (better ER and FF than enrollment criteria), b) 18% (82 patients) had initiated treatment prior to enrollment (PT, injection, …, c) 6% (27 patients) had OA, inflammatory joint disease, full thickness rotator cuff tear, trauma, …, d) 4% (18 patients) had prior surgery on shoulder, e) 1% (5 patients) didn’t get cortisone shot during 1^st^ visit. ** Other reasons: 17% (77 patients) not good candidates for study (patient live too far to regularly commute, non-English speaker, can’t commit long term, leaving state, moving…)

Thirty-four patients (34 shoulders) between the ages of 38–74 diagnosed with AC were enrolled in the study (Fig. 1). Patients were randomized into one of three groups: HIS device only (*n* = 13), PT only (*n* = 10), and combined PT + HIS device (*n*= 11). Adhesive capsulitis was defined as shoulder pain with limited ROM for more than one month with ≤ 130 degrees of passive forward flexion and ≤ 30 degrees of passive external rotation [16]. A minimum one-month criterion was selected to exclude all patients with short-term, temporary loss of motion which could have been attributed to causes unrelated to AC. Patients with prior treatment of the involved shoulder, including previous injections or PT for AC, shoulder surgery, ipsilateral shoulder infection, or rheumatoid arthritis were excluded. In addition, patients with any disorder that could result in pain or limited ROM such as inflammatory joint disease, osteoarthritis evidenced on radiographs, full-thickness rotator cuff tears identified on ultrasound or MRI imaging, history of trauma (fracture) involving the shoulder, or other shoulder deformity were also excluded. Patients with cognitive deficit, inability to comprehend PT/HIS-device instructions or non-english speakers were also excluded. The surgeon confirmed the presence of AC as defined above before consenting the patients for enrollment. Ethical approval was obtained from the Institutional Review Board. Furthermore, a minimum of 12 months clinical follow-up was required for inclusion in this report. Lastly, patients enrolled in this study received no prior surgical intervention and conservative treatment was selected as the appropriate choice of treatment by the treating surgeon.

### Procedure

A goniometer was used to measure and record passive ROM of both the non-affected and affected shoulder in forward flexion (FF), abduction (ABD), and external rotation (ER). Internal rotation was reported as a numerical value from 0 to 8 for the highest point the patient can reach behind the back: ipsilateral hip (0), ipsilateral back pocket (1), contralateral back pocket (2), S1 to L5 (3), T11 to L1 (4), T7 to T10 (5), T4 to T6 (6), T2 to T3 (7), and C8 to T1 (8) [24]. Each patient was given an intra-articular injection via the Neviaser portal technique consisting of 2 cc 1% lidocaine, 2 cc 0.25% Marcaine, and 40 mg of Kenalog. Injections were given before initiating study treatment. Each patient was assigned to one of three groups by a research staff member using a random number generator: group I used the HIS device alone, group II used physical therapy only, and group III used the HIS device and physical therapy in combination. Patient would start with PT typically within a week of injection. The treating surgeon was unaware of the patient’s treatment group assignment until follow-up. Treating surgeons and physical therapists have over 10 + years of experience.

The PT protocol consisted of shoulder range of motion exercises, including joint mobilization and scapular stabilization as deemed appropriate by the treating physical therapist according to a standardized protocol (Appendix A). Physical therapists instructed patients on proper techniques and specific stretches and exercises. The patients were scheduled for three 60-minute PT sessions per week. Patients continued physical therapy until the affected shoulder achieved external rotation and forward flexion ROM equal to or greater than 90% of the contralateral unaffected side.

**Fig. 2:**
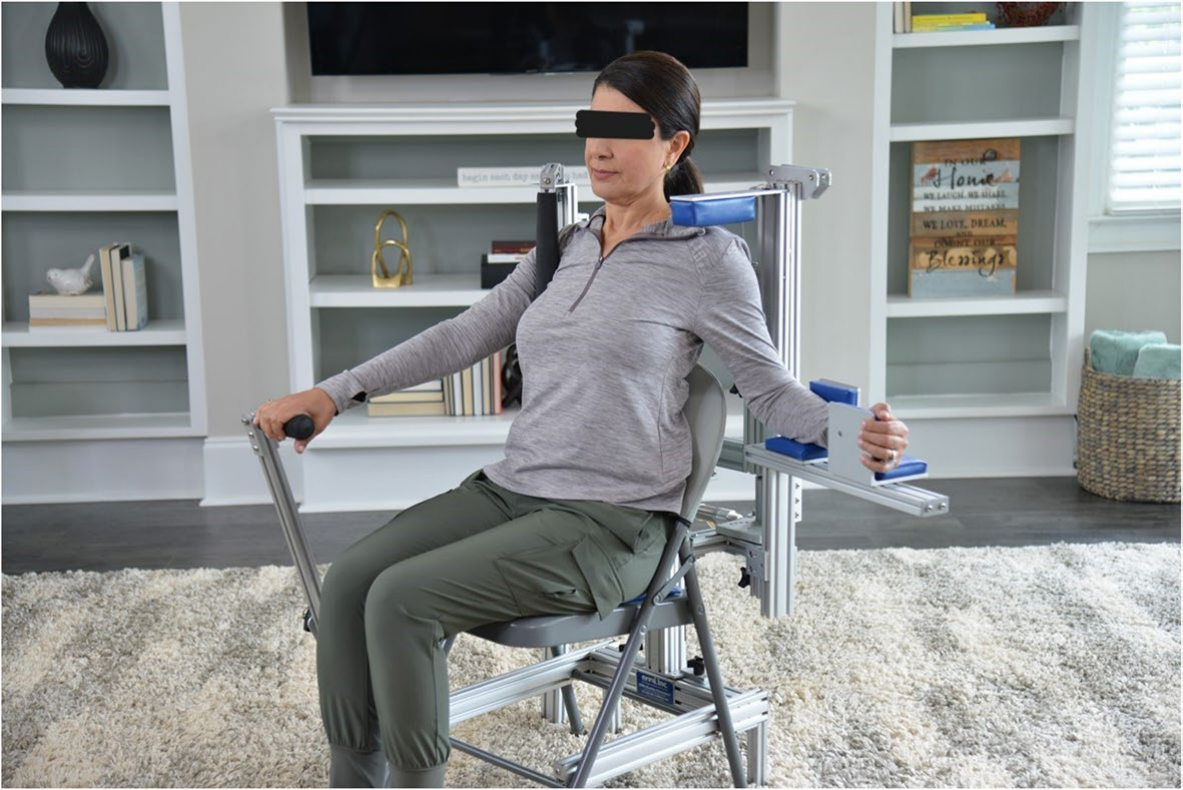
Flexionater

 Patients randomized to the HIS stretch device group (Flexionater Chair, Ermi, Atlanta, GA, Fig. 2) were instructed to stretch at a high intensity for 60 min per day divided into 3 time periods by the representative of the company. Patients were asked to use the HIS for 10 min, followed by 10 min of rest and another 10 min of stretch. This cycle was repeated two more times each day to achieve the goal of 60 min of stretching per day. The chair was initially adjusted for ER. Once 90% of contralateral motion was completed, the HIS stretch device was then changed to perform abduction. The patient continued use until they reached ER and FF of at least 90% of the contralateral side.

Patients in the combined therapy group were instructed to perform the daily HIS stretching exercises while attending PT for 2–3 sessions per week using the exact protocols followed by the other two groups.

### Outcome measures

Primary outcome measures included shoulder range of motion (passive shoulder forward flexion, external rotation, abduction, internal rotation) measured by the treating physician, American Shoulder and Elbow Surgeons standardized shoulder assessment form (ASES) scores for pain and function, and Simple shoulder test (SST) scores [24, 25]. Passive ROM was chosen to isolate gleno-humeral motion and reduce other potential confounding variables. Measurements in forward flexion and external rotation were included in this study as these are standard measurements used by clinicians to assess patient recovery [26]. Furthermore, previous research has shown stretching external rotation and abduction improves the ROM in other planes [16, 22]. Achieving a minimal clinically important difference (MCID) for ASES Total (20 point improvement), and SST (2 point improvement) scores had been determined for each group [27, 28].

Patients in HIS device and combination therapy groups additionally reported on compliance, satisfaction, and convenience with the selected therapy option using simplified 5-point Likert scale. Furthermore, the same patients were asked to self-report their experience with the device (ease of use, comfort, satisfaction, and perception of improvement in shoulder motion) using binary selection (yes/no).

### Statistical analysis

Data analysis was performed using SPSS version 27 (IBM Corp, Armonk, NY). Mean shoulder range of motion, ASES, and SST scores were calculated at baseline, 6 weeks, 3 months, 6 months and the last available follow-up longer than 12 months for subjects in all three groups. A Shapiro Wilcox analysis was used to confirm normality of distribution. A paired t-test was utilized to evaluate improvement from baseline within each group and ANOVA was used to evaluate differences between the groups. Chi-square test was used to evaluate associations between categorical variables. Minimal sample size of 9 per group was determined with calculated effect size of 1.2 (average difference in ER 15 ± 12.5 degrees [16]) power of 80%. Significance was considered at alpha 0.05.

## Results

### Demographics

The average age of the subjects in the study was 56 years (range, 38–74). The average clinical follow up was 18 months (SD, 6.4). Eleven patients (32%) were male. Twenty-one patients had symptoms in the left shoulder (62%), and thirteen patients had symptoms in the right shoulder (38%). Diabetes and thyroid dysfunction were present in 13 patients (38%) and 5 patients (15%), respectively.

There were no statistical differences between study groups in terms of patient’ sex (*p* = 0.497), age (*p* = 0.305), laterality (*p* = 0.4), diagnosis of diabetes (*p* = 0.985) or thyroid dysfunction (*p* = 0.657).

### Treatment efficacy

There were significant improvements in all planes of motion and all recorded patient reported outcome measure (PROM) scores in each of the studied groups from baseline to final follow-up (Table 1, Appendix B).

**Table 1:** Average change from baseline to final follow-up in shoulder ROM and PROMs among three groups

Improvement at min. 1 year	Study Group								
	HIS device + PT			HIS device			PT		
	Mean	SD	*p*-value	Mean	SD	*p*-value	Mean	SD	*p*-value
Forward Flexion (°)	67.3	35.8	< 0.001	70.0	32.0	< 0.001	47.5	25.5	< 0.001
Abduction (°)	68.6	25.9	< 0.001	100.0	32.0	< 0.001	57.8	28.4	< 0.001
External Rotation (°)	56.4	22.0	< 0.001	50.0	39.7	< 0.001	30.0	18.3	< 0.001
Internal Rotation^a^	3.9	1.3	< 0.001	4.3	2.1	< 0.001	3.3	2.2	< 0.001
SST	7.8	2.6	< 0.001	7.5	2.6	< 0.001	6.8	3.6	< 0.001
ASES Pain	32.3	12.9	< 0.001	28.3	16.4	< 0.001	23.3	10.0	< 0.001
ASES Function	27.9	10.9	< 0.001	29.6	9.0	< 0.001	25.2	15.8	< 0.001
ASES Total	60.2	20.1	< 0.001	57.9	20.1	< 0.001	49.1	24.6	< 0.001

*PROM *Patient reported outcome measures, *PT *Physical therapy, *SD s*tandard deviation,  *HIS *High intensity stretching device; ^a^internal rotation is measured based on a scale (0–8) representing the patient’s ability to reach anatomic landmarks posteriorly. Zero represents the least amount of internal rotation (ipsilateral hip), and 8 represents the most (C8 - T1)

The HIS group achieved a minimum of 95% of contralateral shoulder ROM in all planes by final follow-up. Combination therapy achieved a minimum of 92% while PT alone achieved minimum of 82% of contralateral motion (Table 2).

**Table 2:** Percentage of contralateral ROM at baseline and final follow-up among three groups

	Study Group					
	HIS device + PT		HIS device		PT	
	Baseline	at min. 1 year	Baseline	at min. 1 year	Baseline	at min. 1 year
Percent of contralateral Forward Flexion	54%	96%	56%	97%	62%	93%
Percent of contralateral Abduction	52%	95%	39%	110%	57%	93%
Percent of contralateral External Rotation	-1%	96%	27%	95%	31%	82%
Percent of contralateral Internal Rotation	23%	92%	17%	101%	32%	106%

*ROM *Range of motion, *PT *Physical therapy, *HIS *High intensity stretching device

All patients in the HIS and PT + HIS groups reached MCID for ASES, while 75% of patients in PT group achieved similar improvement. All patients in the HIS and PT + HIS device groups reached MCID for SST compared to 88% of patients in the PT group.

### Rate of improvement

**Fig. 3:**
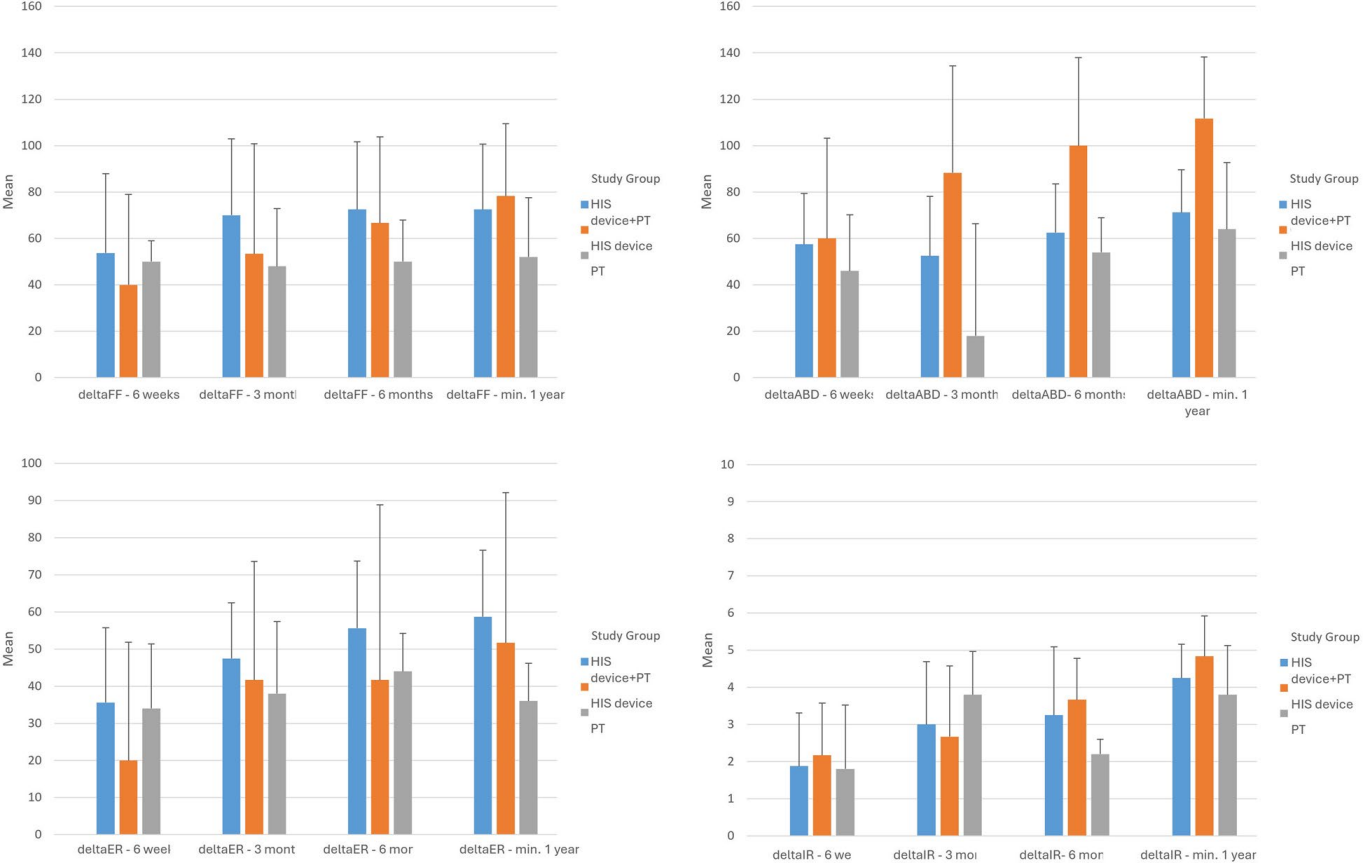
Comparison of change in shoulder ROM among the three groups. Bars represent standard error. Delta values are calculated by subtracting value at the studied follow-up from its baseline

**Fig. 4:**
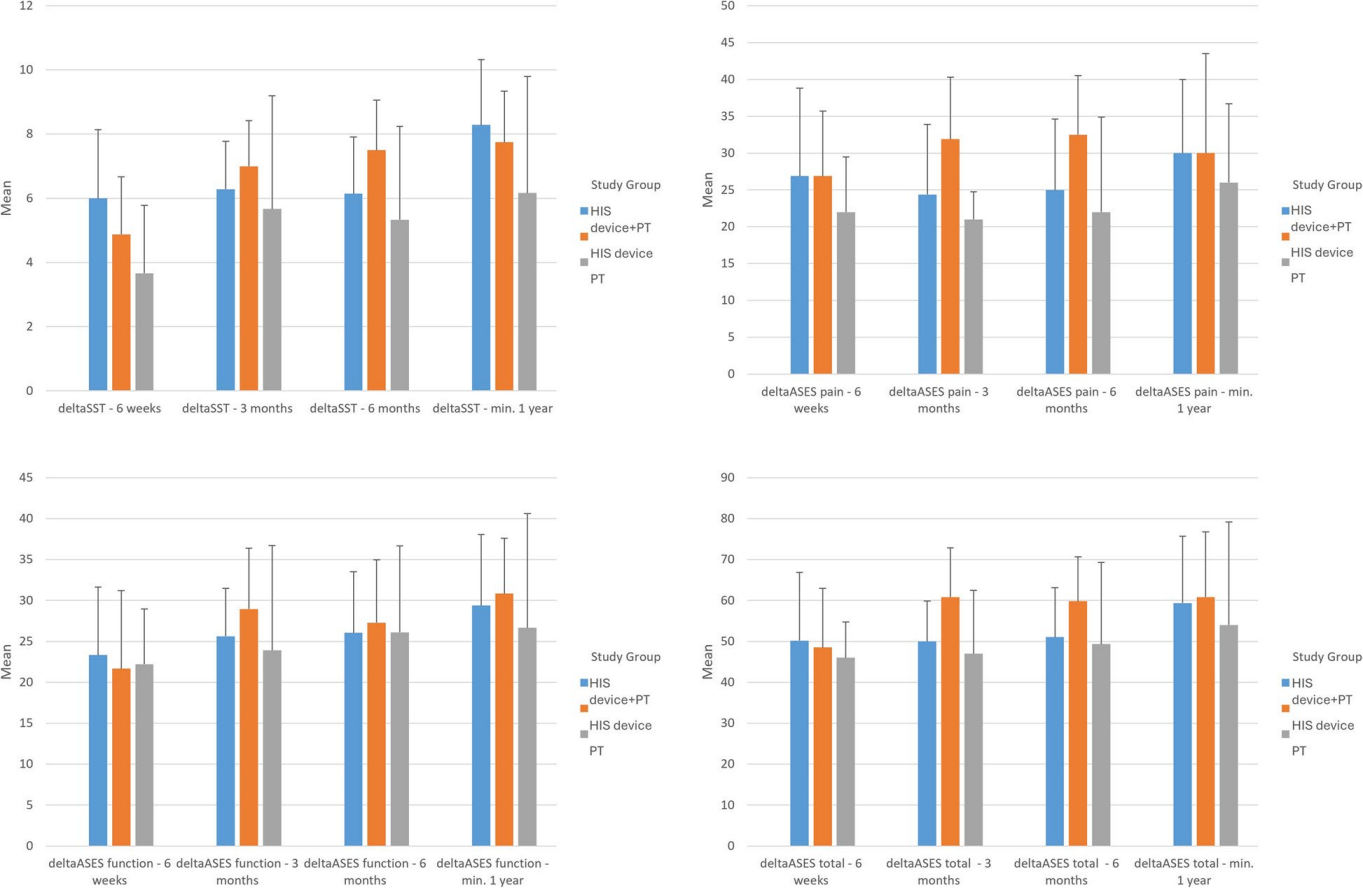
Comparison of change in PROMs among the three groups. Bars represent standard error. Delta values are calculated by subtracting value at the studied follow-up from its baseline

HIS device overperformed PT in abduction as early as in 3 months (*p* = 0.04), 6 months (*p* = 0.028), and final follow up (*p* = 0.003 for HIS device alone, Fig. 3, Appendix C). Moreover, HIS device showed significantly larger improvement in SST score (*p* = 0.045) and ASES Total score (*p* = 0.048) at 6 months when compared to PT (Fig. 4, Appendix D).

### Compliance and satisfaction

Compliance, satisfaction, and convenience with the HIS device was recorded via patient questionnaire. Compliance was self-reported, with 67% (HIS device group) and 85% (HIS device group + PT) of patients reported using HIS-device exactly as prescribed or more, respectively. Remaining 33% of HIS device group patients and 14% HIS device + PT group patients used HIS device less than prescribed. Patients reported greater satisfaction (100% very satisfied in HIS device, 57% very satisfied and 43% satisfied in HIS device + PT) and convenience (92% very convenient and 8% convenient with HIS device compared to 71% very convenient and 29% convenient with HIS device + PT) of treatment plan with HIS device alone versus in combination with in-person physical therapy. The majority of patients reported comfort and ease of the HIS device (95% found HIS device easy to use, 84% found it comfortable, and 95% easy to perform repetitions) and 95% patients reported that HIS served as an effective means to restore ROM, and 100% was satisfied with improvement in shoulder motion.

Three patients (30%) in the PT group required additional injection beyond 6 months follow-up compared to no patients in the HIS group and only 1 patient (9%) in the combination group.

## Discussion

Adhesive capsulitis of the shoulder is a painful and disabling disease. Although physical therapy alone can be effective [3, 9, 10], it can be associated with substantial financial cost and time burden [16]. As patients using the HIS device had increased rate of recovery and decreased levels of pain, they are likely able to return to work faster, as well as require fewer follow-up clinician visits and procedures. Therefore, effective treatment of patients with AC using an at-home HIS device could result in significant savings for the healthcare system and for patients. The HIS device evaluated in this study presents a viable alternative to PT when PT is unavailable, which may facilitate greater patient compliance and better outcomes.

This study showed that patients using a HIS device to treat AC may achieve better motion than patients enrolled in traditional PT (Fig. 3) to nearing motion seen in the unaffected contralateral shoulder. Additionally, a higher percentage of patients using the HIS device met ASES and SST MCID thresholds than patients treated with PT alone. Furthermore, neither traditional PT nor combination therapy proved to be significantly better than the HIS device in any reported outcomes. Although not specifically studied here, we suspect that compliance with HIS device use may be a factor explaining this finding. As the HIS device is set up in the patient’s home, patients may find it easier to use the device and complete their prescribed treatment time. During the course of this study, the COVID-19 epidemic was an additional factor that may have limited patient’s ability to maintain a consistent therapy regimen, as reflected by large withdrawals from the study by patients in both PT and HIS + PT groups.

The effectiveness of static stretching devices has been established in the literature for extremities other than the shoulder [29–31]. It has been theorized that prolonged, static stretching leads to permanent, progressive elongation of collagen fibers present in the inflammatory reactive tissues that cause the contracture inherent to adhesive capsulitis [32, 33]. Very few studies have evaluated a HIS device for primary non-operative treatment of AC. The results of the current paper are supported by Ibrahim et al., who evaluated the use of a static progressive stretch device coupled with physical therapy in the treatment of AC [17]. This prospective, randomized controlled trial consisted of 60 patients with a mean follow-up of 1 year. Patients were randomized to traditional physical therapy or both traditional physical therapy and a static stretching device concurrently. At final follow-up, the authors found a significantly greater increase in all ROM scores in the combination group compared to the PT alone group. Additionally, VAS pain scores were significantly lower in the combination group. The authors concluded that a static stretching device should be added to traditional PT in the treatment of AC. The results of our study also found greater final ROM measurements in the combined group compared to the traditional PT group. The current study differs from Ibrahim et al.‘s in that we included a group who used the HIS device without PT, which allowed the direct comparison between the HIS device and PT. We found greater average improvements after 6 weeks in FF, abduction, ER, SST scores, and ASES Pain, Function and Total scores in the HIS device compared to the PT group.

In addition to loss of motion, adhesive capsulitis is known to be a significantly painful condition. Corticosteroid injection is a common means of pain control for AC and was provided to all patients at the initial visit. 30% of patients in the PT group required additional injection beyond 6 months follow-up compared to no patients in the HIS group and only 1 patient in the combination group. Patients in the HIS device and combination groups showed better maintenance of pain control even at 6 months, in comparison to the PT only group. These results may in turn also explain the improved maintenance of range of motion in the HIS device and combination groups in comparison to the PT only group, however, larger cohort would be needed to fully evaluate all confounding effects.

This study does have several limitations. First, some early participants in the study did not have complete follow-up due to COVID-19 related protocol deviations (particularly occurring for patients randomized to PT group (*n* = 7) and combined HIS device and PT groups (*n* = 7)) and were withdrawn from the study. Despite being blinded before randomization, the treating surgeon was aware of the randomized group at follow-up visits. This was done to ensure and record compliance with assigned treatments.

## Conclusion

In this randomized controlled trial, it was found that the HIS stretch device studied was as good or better in every outcome recorded, including ROM (FF, ER, Abd, and IR), ASES, and SST when compared to PT alone or in combination with PT. The at-home HIS device studied may be an option for first-line therapy in treating patients diagnosed with adhesive capsulitis.

### Supplementary Information

**Supplementary Material 1.**

**Supplementary Material 2.**

**Supplementary Material 3.**

**Supplementary Material 4.**

## Data Availability

The datasets used and analyzed during the current study are not publicly available due to lack of participant consent to share their data but are available from the corresponding author upon reasonable request and after ethical considerations are met.
