# Supplementary material for: Efficacy of a high-intensity home stretching device and traditional physical therapy in non-operative management of adhesive capsulitis - a prospective, randomized control trial
Source: BMC Musculoskelet Disord. 2024 Apr 20;25:305. doi: 10.1186/s12891-024-07448-4 (PMC11031861; doi:10.1186/s12891-024-07448-4)
Supplement: Supplementary file 1 — Supplementary Material 1. [file 12891_2024_7448_MOESM1_ESM.docx]

Appendix A: PT protocol

**Comprehensive Rehabilitation Protocol for Patients with Adhesive Capsulitis**

For patients at any stage of adhesive capsulitis development:

Suggested Treatment Strategies: (at discretion of the treating Physical Therapist)

- Modalities used: Thermotherapy (Heat/Ice) and E-stim (TENS) units as needed for pain
- Correction of posture and biofeedback: scapular retraction, GH isolation, decrease compensation with exercises done in front of mirror to ensure correct form
- PROM/AROM/AAROM as follows/tolerated:
  - Rope & Pulley Flexion
  - Supine forward flexion using L bar
  - Abduction
  - Internal and external rotation
  - Internal and external rotation at 90 degrees abduction
  - Bent-over, light weighted pendulums
  - End range GH-stretching via L bar, doorways, or physical therapist
    - 20-30 seconds or as tolerated
    - Target emphasis on patient deficiency
  - Light isometric strengthening
  - Light free weight strengthening
  - Shoulder/scapular stabilization strengthening
    - Emphasis on recruitment of rhomboids, middle trapezius, and serratus anterior as applies
- Ten minutes ice application for pain
- NSAIDs PRN
- May inquire with treating physician for CS injection

Goals and Focuses:

- Maximize TERT as possible to take advantage of plastic deformation principles
- Begin with passive->AROM->AAROM to end range of motion progressively
- Improve GH volume, compliance, elasticity
- Improve strength in conjunction with ROM
- Normalize postural deficiencies, decrease compensatory movements (i.e. scapular elevation due to loss of abduction capability)
- Encourage home exercises with handout and low weight-bearing, high-ROM activities (i.e. swimming, golf swings, pendulums, wall crawls, door way stretching)
