# Supplementary material for: Efficacy of a high-intensity home stretching device and traditional physical therapy in non-operative management of adhesive capsulitis - a prospective, randomized control trial
Source: BMC Musculoskelet Disord. 2024 Apr 20;25:305. doi: 10.1186/s12891-024-07448-4 (PMC11031861; doi:10.1186/s12891-024-07448-4)
Supplement: Supplementary file 2 — Supplementary Material 2. [file 12891_2024_7448_MOESM2_ESM.docx]

Appendix B: Additional details on baseline (a) and final outcomes (b)

| (a) ROM & PROMs at baseline | Study Group | | | | | |
| --- | --- | --- | --- | --- | --- | --- |
|  | HIS device + PT | | HIS device | | PT | |
|  | Mean | SD | Mean | SD | Mean | SD |
| Forward Flexion (°) | 96.4 | 45.4 | 100.9 | 25.9 | 111.0 | 14.5 |
| Abduction (°) | 89.1 | 25.5 | 69.1* | 23.9 | 99.0* | 25.6 |
| External Rotation (°) | 10.0 | 21.0 | 22.7 | 26.5 | 22.0 | 9.2 |
| Internal Rotation ^▲^ | 1.4 | 1.7 | 1.0 | 0.8 | 1.9 | 1.9 |
| SST | 3.5 | 2.3 | 2.9 | 2.4 | 3.0 | 2.5 |
| ASES Pain | 12.3 | 11.3 | 12.5 | 9.7 | 15.0 | 9.0 |
| ASES Function | 18.5 | 11.6 | 15.8 | 8.6 | 16.5 | 10.9 |
| ASES Total | 30.8 | 19.4 | 28.3 | 12.6 | 30.4 | 14.2 |

* p=0.01

| (b) ROM & PROMs at min. 1 year | Study Group | | | | | |
| --- | --- | --- | --- | --- | --- | --- |
|  | HIS device + PT | | HIS device | | PT | |
|  | Mean | SD | Mean | SD | Mean | SD |
| Forward Flexion (°) | 163.6 | 26.9 | 166.7 | 15.6 | 158.5 | 13.8 |
| Abduction (°) | 157.7 | 28.8 | 165.8 | 12.4 | 156.8 | 23.6 |
| External Rotation (°) | 66.4 | 24.6 | 72.5* | 17.1 | 52.0* | 18.1 |
| Internal Rotation ^▲^ | 5.3 | 1.3 | 5.0 | 2.0 | 5.2 | 1.3 |
| SST | 11.3 | 2.2 | 10.5 | 2.4 | 9.8 | 2.0 |
| ASES Pain | 44.5 | 9.1 | 41.2 | 11.9 | 39.0 | 12.0 |
| ASES Function | 46.4 | 6.7 | 45.5 | 6.3 | 41.7 | 6.6 |
| ASES Total | 90.9 | 15.5 | 86.7 | 16.8 | 80.7 | 17.8 |

* p=0.024

PROM, patient reported outcome measures; ROM, range of motion PT, physical therapy; SD, standard deviation; HIS, high intensity stretching device; ^▲^ internal rotation is measured based on a scale (0-8) representing the patient's ability to reach anatomic landmarks posteriorly. Zero represents the least amount of internal rotation (ipsilateral hip), and 8 represents the most (C8 - T1).
