# Supplementary material for: Efficacy of a high-intensity home stretching device and traditional physical therapy in non-operative management of adhesive capsulitis - a prospective, randomized control trial
Source: BMC Musculoskelet Disord. 2024 Apr 20;25:305. doi: 10.1186/s12891-024-07448-4 (PMC11031861; doi:10.1186/s12891-024-07448-4)
Supplement: Supplementary file 3 — Supplementary Material 3. [file 12891_2024_7448_MOESM3_ESM.docx]

Appendix C: Additional details on ROM throughout treatment period

| Improvement | | Study Group | | | | | |
| --- | --- | --- | --- | --- | --- | --- | --- |
|  |  | HIS device + PT | | HIS device | | PT | |
|  |  | Mean | SD | Mean | SD | Mean | SD |
| Forward Flexion (°) | 6 weeks | 47.3 | 43.6 | 38.0 | 37.1 | 39.4 | 25.3 |
|  | 3 months | 68.9 | 43.7 | 50.0 | 46.9 | 42.9 | 29.8 |
|  | 6 months | 72.2 | 38.7 | 68.0 | 34.9 | 47.5 | 15.8 |
|  | min. 1 year | 67.3 | 35.8 | 70.0 | 32.0 | 47.5 | 25.5 |
|  |  |  |  |  |  |  |  |
| Abduction (°) | 6 weeks | 43.6 | 48.8 | 54.0 | 44.8 | 31.7 | 32.0 |
|  | 3 months | 55.6 | 35.0 | 75.6* | 50.3 | 25.7* | 50.3 |
|  | 6 months | 65.6 | 29.2 | 89.0^ | 50.0 | 47.5^ | 24.3 |
|  | min. 1 year | 68.6^⁂^ | 25.9 | 100.0^†⁂^ | 32.0 | 57.8^†^ | 28.4 |
|  |  |  |  |  |  |  |  |
| External Rotation (°) | 6 weeks | 34.1 | 33.4 | 34.0 | 34.7 | 18.9 | 26.7 |
|  | 3 months | 47.8 | 19.9 | 41.1 | 32.2 | 34.3 | 20.7 |
|  | 6 months | 54.2 | 24.3 | 47.0 | 45.7 | 35.0 | 16.0 |
|  | min. 1 year | 56.4^∆^ | 22.0 | 50.0 | 39.7 | 30.0^∆^ | 18.3 |
|  |  |  |  |  |  |  |  |
| Internal Rotation ^▲^ | 6 weeks | 2.1 | 1.8 | 2.1 | 1.5 | 1.6 | 2.2 |
|  | 3 months | 3.3 | 2.4 | 2.7 | 2.0 | 3.1 | 1.8 |
|  | 6 months | 3.2 | 2.4 | 3.7 | 1.6 | 1.9 | 1.6 |
|  | min. 1 year | 3.9 | 1.3 | 4.3 | 2.1 | 3.3 | 2.2 |

* p=0.04; ^ p=0.028; ^†^p=0.003, ^⁂^p=0.019, ^∆^p=0.04

PROM, patient reported outcome measures; ROM, range of motion PT, physical therapy; SD, standard deviation; HIS, high intensity stretching device; ^▲^ internal rotation is measured based on a scale (0-8) representing the patient's ability to reach anatomic landmarks posteriorly. Zero represents the least amount of internal rotation (ipsilateral hip), and 8 represents the most (C8 - T1).
