# Supplementary material for: Efficacy of a high-intensity home stretching device and traditional physical therapy in non-operative management of adhesive capsulitis - a prospective, randomized control trial
Source: BMC Musculoskelet Disord. 2024 Apr 20;25:305. doi: 10.1186/s12891-024-07448-4 (PMC11031861; doi:10.1186/s12891-024-07448-4)
Supplement: Supplementary file 4 — Supplementary Material 4. [file 12891_2024_7448_MOESM4_ESM.docx]

Appendix D: Additional details on PROMs throughout treatment period

| Improvement | | Study Group | | | | | |
| --- | --- | --- | --- | --- | --- | --- | --- |
|  |  | HIS device + PT | | HIS device | | PT | |
|  |  | Mean | SD | Mean | SD | Mean | SD |
| SST | 6 weeks | 5.2 | 2.5 | 4.1 | 2.8 | 3.4 | 2.5 |
|  | 3 months | 6.1 | 1.9 | 6.7 | 1.9 | 5.4 | 4.1 |
|  | 6 months | 6.0 | 2.4 | 7.5* | 2.0 | 5.0* | 3.5 |
|  | min. 1 year | 7.8 | 2.6 | 7.5 | 2.6 | 6.8 | 3.6 |
|  |  |  |  |  |  |  |  |
| ASES Pain | 6 weeks | 25.5 | 17.5 | 25.0 | 12.2 | 18.1 | 9.2 |
|  | 3 months | 26.7 | 14.4 | 29.5 | 11.9 | 20.0 | 8.2 |
|  | 6 months | 25.6 | 12.9 | 30.5 | 10.4 | 19.3 | 12.7 |
|  | min. 1 year | 32.3 | 12.9 | 28.3 | 16.4 | 23.3 | 10.0 |
|  |  |  |  |  |  |  |  |
| ASES Function | 6 weeks | 22.0 | 10.5 | 19.3 | 12.9 | 22.2 | 10.0 |
|  | 3 months | 26.1 | 7.9 | 26.0 | 11.3 | 23.1 | 16.3 |
|  | 6 months | 25.6 | 10.0 | 28.3 | 9.7 | 21.9 | 14.0 |
|  | min. 1 year | 27.9 | 10.9 | 29.6 | 9.0 | 25.2 | 15.8 |
|  |  |  |  |  |  |  |  |
| ASES Total | 6 weeks | 47.4 | 23.6 | 44.3 | 20.2 | 41.5 | 15.0 |
|  | 3 months | 52.8 | 15.5 | 55.5 | 18.8 | 44.5 | 22.7 |
|  | 6 months | 51.1 | 16.0 | 58.8^ | 14.0 | 41.4^ | 22.9 |
|  | min. 1 year | 60.2 | 20.1 | 57.9 | 20.1 | 49.1 | 24.6 |

* p=0.045; ^ p=0.048

PROM, patient reported outcome measures; PT, physical therapy; SD, standard deviation; HIS, high intensity stretching device.
